# Detecting and Quantifying Mind Wandering during Simulated Driving

**DOI:** 10.3389/fnhum.2017.00406

**Published:** 2017-08-08

**Authors:** Carryl L. Baldwin, Daniel M. Roberts, Daniela Barragan, John D. Lee, Neil Lerner, James S. Higgins

**Affiliations:** ^1^Department of Psychology, George Mason University Fairfax, VA, United States; ^2^Department of Industrial and Systems Engineering, University of Wisconsin-Madison Madison, WI, United States; ^3^Center for Transportation, Technology and Safety Research, Westat Rockville, MD, United States; ^4^Office of Behavioral Safety Research, National Highway Traffic Safety Administration, U.S. Department of Transportation Washington, DC, United States

**Keywords:** mind wandering, inattention, driving, EEG, alpha

## Abstract

Mind wandering is a pervasive threat to transportation safety, potentially accounting for a substantial number of crashes and fatalities. In the current study, mind wandering was induced through completion of the same task for 5 days, consisting of a 20-min monotonous freeway-driving scenario, a cognitive depletion task, and a repetition of the 20-min driving scenario driven in the reverse direction. Participants were periodically probed with auditory tones to self-report whether they were mind wandering or focused on the driving task. Self-reported mind wandering frequency was high, and did not statistically change over days of participation. For measures of driving performance, participant labeled periods of mind wandering were associated with reduced speed and reduced lane variability, in comparison to periods of on task performance. For measures of electrophysiology, periods of mind wandering were associated with increased power in the alpha band of the electroencephalogram (EEG), as well as a reduction in the magnitude of the P3a component of the event related potential (ERP) in response to the auditory probe. Results support that mind wandering has an impact on driving performance and the associated change in driver’s attentional state is detectable in underlying brain physiology. Further, results suggest that detecting the internal cognitive state of humans is possible in a continuous task such as automobile driving. Identifying periods of likely mind wandering could serve as a useful research tool for assessment of driver attention, and could potentially lead to future in-vehicle safety countermeasures.

## Introduction

Driver inattention is a frequent cause of automobile crashes and fatalities. This issue has received considerable attention from the scientific community in recent years. Methods of detecting episodes of driver inattention in real-time hold promise for alleviating the human and economic costs of this safety critical issue. Drivers can be inattentive for a variety of reasons, the most obvious being distraction from mobile devices (Caird et al., 2008) or other external factors. However, many distracted driving crashes occur in the absence of an obvious visual or manual distraction. Mind wandering has been suggested as a potential source of many of these distracted driving crashes.

For most people, driving is a highly-overlearned task. Consequently, many of the tasks of everyday driving—lane and speed maintenance, stopping at signaled intersections, etc.—tend to occur relatively automatically. In addition, many trips are routinized with drivers taking the same routes back and forth to work, the grocery store, or other frequently visited locations, which further promotes automaticity, allowing attention to be devoted to other activities. The routine nature of the driving task, particularly along familiar or monotonous routes, creates an environment ripe for internal distraction or mind wandering, as we will refer to it here. Nevertheless, to maintain safety, drivers must remain attentive to a wide variety of stimuli that may represent latent hazards (Fisher et al., 2002) and be able to swiftly and accurately respond to unexpected events.

There are many varieties of attention (Parasuraman, 1998), but a well-accepted theoretical distinction is between external and internal attention (Chun et al., 2011). External (or “bottom-up”) attention is triggered reflexively by environmental events. Internal (or “top-down”) attention is voluntary or involuntary application of cognitive resources away from the external environment towards internal thoughts. Most empirical research on internal attention has investigated the pursuit of goals relevant to events in the environment—e.g., such as searching for a landmark while driving in an unfamiliar neighborhood. But, by definition, internal attention can also be devoted to thoughts and memories quite unrelated to any external event. Much less research has been devoted to this topic (Forster and Lavie, 2014), but in recent years a small but growing body of work has examined an aspect of such internal attention—mind wandering (Giambra, 1995; Smallwood and Schooler, 2006).

It is well documented that driving performance is modulated by factors such as effort, fatigue and time on task (Robertson et al., 1997; Grier et al., 2003; Helton and Warm, 2008; May and Baldwin, 2009; Langner et al., 2010; Baldwin et al., 2014). Furthermore, several studies have shown that external distractions, such as talking or texting on a mobile phone, impair driving performance (Strayer et al., 2003; Strayer and Drews, 2007; Caird et al., 2008). Less appreciated and understood is the threat to safety posed by mind wandering behind the wheel, though mind wandering has been associated with an increased risk of being responsible for an automobile crash (Galéra et al., 2012). Fatigue associated with increased time on task may exacerbate both the prevalence and potential risk associated with mind wandering as it is associated with withdrawal of attention away from the driving task and can be considered a form of internal distraction (Williamson, 2007).

Mind wandering has been defined as, “a shift of attention away from a primary task toward internal information” (Smallwood and Schooler, 2006, p. 946). It often occurs without intention and may even occur without explicit awareness, making it a particular challenge to observe and measure. People may continue to move their eyes across a page of text (or the forward field of view while driving) without overtly attending to the viewed stimuli (Smallwood, 2011). Mind wandering is associated with increased activity in the default mode network (DMN) and decreased activity in the dorsal attention network (DAN). The DAN is integrally involved in controlling eye movements and directing exogenous attention to salient stimuli through top-down goal directed processing (Carretié et al., 2013). The DMN is sensitive to the presence of biologically salient non-task relevant stimuli, but this sensitivity is generally thought to come at the cost of processing task-relevant stimuli (Smallwood, 2011; Carretié et al., 2013). Smallwood (2011) has referred to this interplay between the DAN and DMN as a decoupling process, meaning that as attention is shifted toward one system it is withdrawn from the other. This decoupling may have important implications for driving by suggesting that the more drivers are engaged in mind wandering (activation of the DMN) the less likely they are to process external perceptual cues (potential hazards), particularly if the perceptual cues involve non-biologically salient stimuli (e.g., artificial sounds such as alarms and brake lights).

Two methods have typically been used to detect and quantify mind wandering: self-caught detection and probe-caught detection. The self-caught method of detection involves participants reporting when they notice their mind wandering. In contrast to self-caught methods of detection, probe techniques allow for sampling participants cognitive states throughout a task under experimenter control, thus allowing for the detection of mind wandering episodes that are uncaught by the participants themselves. Three types of probe techniques have been previously utilized. The first and most commonly used is intermittent presentation of questions such as, “Just now, were you mind wandering?” throughout an otherwise continuous task (e.g., Broadway et al., 2015). Another variation of this technique uses probe tones, prompting participants to indicate via a button press whether they were or were not mind wandering (Smallwood and Schooler, 2006). More recently, Seli et al. (2016) used a combination of these probe techniques to determine whether performance differs when participants were aware or unaware that they were mind wandering. Participants were asked to indicate whether their thoughts were on task or they were mind wandering just before they heard the probe tone. Additionally, if participants indicated that they were mind wandering, they were then asked to indicate if they were aware or unaware that they were mind wandering prior to the probe tone. The third probe-caught method involves participants indicating the content of their thoughts at the time of probe, leaving the experimenter to classify whether their thoughts constituted mind wandering (Smallwood and Schooler, 2006). However, by necessity, both self-caught and probe-caught methods likely disrupt participants primary task performance. This may be especially true when probes require more fine-grained judgments of the degree of mind wandering. An ideal mind wandering detection methodology would not require any response on the part of the participant, but rather would provide an on-line classification using some form of machine learning. However, currently such a technique awaits further exploration of the sensitivity and robustness of various metrics using some type of self-report technique.

Results of several recent studies utilizing measures derived from electroencephalography (EEG) substantiate this claim by observed reductions in perceptual sensitivity during periods of mind wandering (Smallwood et al., 2008; Braboszcz and Delorme, 2011). In a breath counting task, Braboszcz and Delorme (2011) report that in the 10 s prior to participants’ self-detecting a state of mind wandering, there was a significant reduction of alpha band activity combined with a diffuse increase in theta band activity, relative to the 10 s period following the button press, during which participant thought had presumably returned to the breath counting task. Modulation of theta band activity may provide a means of distinguishing mind wandering from other types of internal distractions. Savage et al. (2013) found that when participants were given a riddle to solve while performing a simulated driving task there was a similar increase in theta band as that reported by a study measuring frontal sites during mind wandering (Braboszcz and Delorme, 2011). However, in contrast to mind wandering, being internally distracted by a secondary cognitive task leads to a decrease in theta band activity over occipital electrode locations.

Oscillatory activity in the alpha band of the EEG is suggested to be related to attention processes (Klimesch, 2012), specifically the degree to which attention is allocated internally vs. externally. For example, greater alpha power at parietal and/or occipital electrode locations is associated with the failure to detect (Ergenoglu et al., 2004) or discriminate (Van Dijk et al., 2008; Roberts et al., 2014) visual stimuli, while spatial attention processes similarly modulate alpha power such that alpha is suppressed contralaterally and enhanced ipsilaterally to the attended location (Worden et al., 2000; Thut et al., 2006). In contrast, alpha power has been reported to be elevated for tasks in which attention is directly internally, such as working memory retention (Jensen et al., 2002). As periods of high alpha power are associated with lapses of attention to external stimuli (O’Connell et al., 2009; Zauner et al., 2012; Borghini et al., 2014) alpha power is expected to be greater during periods of mind wandering relative to periods of on task behavior.

In terms of event related potentials (ERPs), modulations of early perceptual and attentional components during mind wandering have been observed. Broadway et al. (2015) report that in a reading task the visual N1 component, thought to index orienting and enhancement of perceptual information (Hopfinger et al., 2004), was attenuated during mind wandering. The authors also report an attenuation of the P1 component, thought to index the inhibition of irrelevant information (Hopfinger et al., 2004), over the right hemisphere when participants reported mind wandering. Attenuation of the visual P1 was reported by Baird et al. (2014) bilaterally. However, it should be noted that such early components are relatively small and short-lasting and thus may be difficult to utilize in terms of on-line detection of mind wandering, especially.

More promising as a means of future on-line detection of mind wandering is the modulation of longer latency components such as the P300, a large, long-lasting ERP component thought to reflect allocation of attentional resources (Nieuwenhuis et al., 2005). Smallwood et al. (2008) found that the P300 evoked by visual stimuli in the sustained attention to response task (SART) was significantly attenuated during periods of self-reported mind wandering relative to periods of self-reported on-task behavior. Similar P300 attenuation during mind wandering was reported by Kam et al. (2014) in response to evaluation of more complex visual stimuli. The relatively high amplitude of the P300 relative to the background EEG allows reasonable extraction of this waveform from single trials for brain-computer interfaces, and has been an area of significant research during the past decade (Cecotti and Rivet, 2014).

Driver behavioral metrics such as speed variability, lane deviation, standard deviation of lateral position (SDLP) and steering reversal rate (SRR), have been shown to serve as an additional method to detect mind wandering (He et al., 2011, 2014; Bencich et al., 2014; Yanko and Spalek, 2014). However, previous research using these behavioral metrics have found inconsistent results that vary depending on the mind wandering detection method. For example, He et al. (2011) used the self-caught detection method and report that speed variability was decreased during mind wandering relative to on-task states. When using the probe detection method to detect instances of mind wandering, Yanko and Spalek (2014) found that mean speed was greater during mind wandering than on-task states. Conversely, using this same technique, Bencich et al. (2014) found that mean speed and speed variability were reduced during mind wandering compared to a state of alertness.

Further, He et al. (2014) found that under low cognitive load, which is thought to be similar to a mind wandering state, SDLP and SRR were reduced compared to a high cognitive load condition. He et al. (2014) interpreted these findings as suggesting that under high cognitive load, more effort and attention is needed to maintain lateral control, which could reflect an alert state. For example, during an attentive state (relative to mind wandering) participants drove at significantly greater speeds, had greater speed variability, greater SDLP and increased SRR (He et al., 2011; Bencich et al., 2014; Yanko and Spalek, 2014). However, though it is well-understood that mind wandering is associated with decreased attention to a primary task, there is currently no consensus of the effects of mind wandering on driving performance.

The purpose of this research was to investigate the frequency of mind wandering over repeated exposure to the same driving route, as well as to identify the relationship between mind wandering and both driver behavior and electrophysiology.

## Materials and Methods

### Procedure

The current study assessed the relationship between mind wandering and driving across 5 days, with the time of day for participation maintained over days within each participant. While a given participant returned at the same time of day for each of their five sessions, the time of day used between participants varied between the morning and afternoon due to participant scheduling availability. The duration of each experimental session lasted from 2 h to 3 h. Each experimental task (the SART and two simulated drives) took approximately 20 min to complete. All procedures were approved by a University Institutional Review Board of George Mason University (protocol # 727867-5) and participants provided written informed consent in accordance with the Declaration of Helsinki.

The procedures were consistent across the five experimental days with the exception of the first day, which included additional procedures. On day 1 only, participants signed an informed consent form describing the study, performed the Rosenbaum and Snellen visual acuity tests, and completed the demographics and driving history questionnaire, Simulator Sickness Screening, and six additional questionnaires described in “Questionnaires” Section. Additionally, prior to the first experimental drive on the first day of participation, participants completed a 5-min practice drive to familiarize themselves with maneuvering the driving simulator, including steering and braking. As the majority of the questionnaires were expected to be stable across days of participation, participants only completed the KSS and a short questionnaire on sleep quality on days two through five. The remaining procedures were performed across all 5 days.

On each day of participation, a 1-min resting baseline was acquired, during which participants were instructed to relax, keep their eyes open, and to look at the center monitor of the driving simulator. During the baseline, the three driving simulator displays (forward, left and right side screens) displayed visual noise, composed of screen shots of the driving scenario with all pixels randomized. Use of visual noise was used to prevent large changes in screen luminance between the resting baseline and performance of the drive. Participants were then presented with a definition of mind wandering, and were provided a demonstration as to how to respond to the probe tones. The mind wandering definition used in this study, adapted from (Singer and Antrobus, 1972), and Seli et al. (2016), was as follows: “please note that for the purposes of this experiment, the words mind wandering, daydreaming and zoning-out are all synonymous. These are popular terms for which there is no official definition. Despite the subjective nature of the mind wandering experience, we define it as thinking about something unrelated to the immediate task. For example, when driving on a highway it is not unusual for thoughts unrelated to driving to enter your mind. For example, you may think about what you ate for dinner, plans you have later with friends, or an upcoming test. These thoughts are considered mind wandering or off task for the purposes of this experiment whether they occur spontaneously or intentionally. During the experiment, you will periodically hear probe tones. As soon as you hear the tone, please indicate whether you were thinking about the immediate task (either driving or SART) or were mind wandering, meaning you were thinking about something unrelated to the immediate task”.

For both experimental drives, participants were instructed to maintain a speed of 65 miles per hour (MPH), stay in the right lane of the roadway, and keep at least one hand on the steering wheel at all times. Additionally, participants were instructed to indicate on the touchscreen whether their thoughts were on-task or they were mind wandering right before they heard the probe tone by pressing the corresponding buttons. Additionally, if participants indicated that they were mind wandering, a second screen appeared on the touchscreen asking participants to indicate if they were aware or unaware that they were mind wandering before they heard the probe tone. After the first drive was complete, participants completed the SART and then the second driving task.

While the drive was primarily composed of straight highway segments, it also included four curved roadway segments. Due to the potential for differences in attention demand between the straight and curved roadway segments, which could potentially influence attentional state or driving performance, attentional probes that occurred during or within 10 s following a roadway curve were excluded from further analyses.

Although not discussed further, in addition to EEG, participants were also outfitted with a head-mounted (eye-glass) eye-tracker, chest belt heart rate monitor, and were video recorded with a dash board video camera during the experiment.

### Participants

Nine participants (5 men, 4 women) were recruited from George Mason University via an e-mail announcement, and were compensated at a rate of $15 per hour. All participants were at least 18 years of age, had normal or corrected-to-normal vision (verified with Rosenbaum card and Snellen eye chart), and possessed a valid United States driver’s license. On average, participants were 24 years of age (*SD* = 3.37, range: 18–29) and had 8 years (*SD* = 3.19, range: 2–12) of driving experience.

None of the participants were taking any medications known to affect the central nervous system, none of the participants had sustained a major head injury such as a concussion, and all were right handed. None of the participants self-reported experiencing frequent motion sickness, and were additionally screened using the Simulator Sickness Screening (Hoffman et al., 2003) prior to participation. Each participant signed an informed consent document after being briefed on the procedures of the study. Procedures of the study were approved by the George Mason University Institutional Review Board.

### Questionnaires

On the first day of participation, participants completed eight questionnaires: a demographics and driving history questionnaire, Simulator Sickness Screening (Hoffman et al., 2003), Mindful Attention Awareness Scale (MAAS; Brown and Ryan, 2003), Cognitive Failures Questionnaire (CFQ; Broadbent et al., 1982), Mind Wandering Scale (MWS; Singer and Antrobus, 1970; Giambra, 1980), Attention-Related Driving Errors Scale (ARDES; Barragán et al., 2016), Attention-Related Cognitive Errors Scale (ARCES; Cheyne et al., 2006) and Karolinska Sleepiness Scale (KSS; Åkerstedt and Gillberg, 1990). The MAAS, CFQ, MWS, ARDES and ARCES have been shown to reliably predict individuals with a greater propensity of experiencing lapses in attention (Broadbent et al., 1982; Cheyne et al., 2006; Barragán et al., 2016) or mind wandering (Giambra, 1980; Brown and Ryan, 2003; Burdett et al., 2016).

### Driving Task

**Figure 1:**
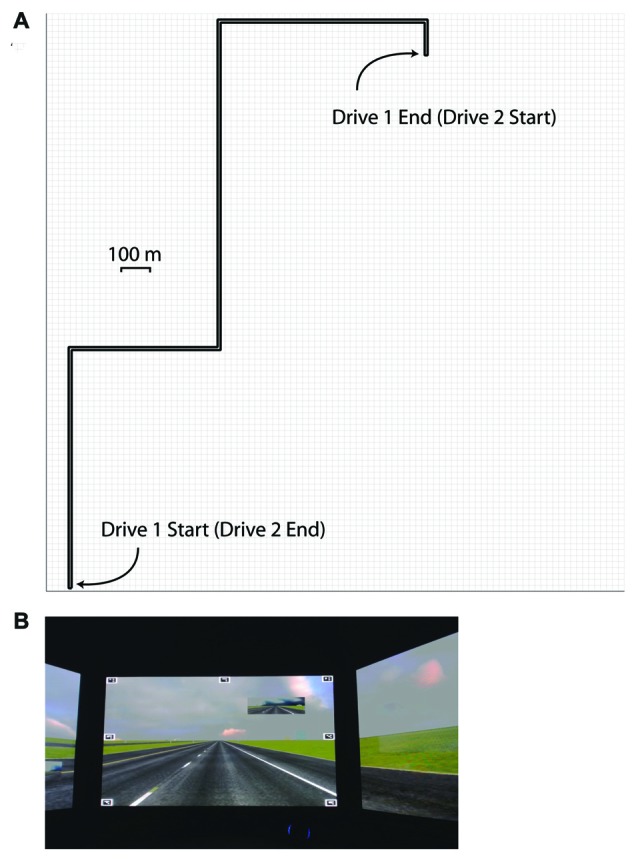
Schematic of the simulated drive **(A)**, and a screen shot of the simulated drive environment **(B)**.

Participants completed two simulated drives on each of the 5 days of participation. The start and end points of the two drives were reversed; on the second drive, the participants began at the destination from the first drive, and drove back to the first drive origin point (see Figure 1).

With the exception of the direction of travel, the driving scenarios were identical between the two drives. The 20 min drives consisted of leaving a parking lot and entering a straight highway with limited scenery and no ambient traffic, until a destination parking lot was reached. The drive did not require navigation; while the highway included curves, participants did not have to make any turns, with the exception of leaving the starting position and entering the destination position. While at the speed limit of 65 MPH, ambient road noise was presented at 60 dB A-weighted sound pressure level (SPL) from speakers integrated into the driving simulator. If participants were driving 15 MPH above or below the speed limit, a message appeared on the center monitor instructing them to slow down or speed up, respectively. Additionally, as participants approached the end of each drive, an auditory message was presented, which instructed the participants to turn into the destination parking lot.

### SART

Between the first and second drives on each day of participation, participants completed the SART (Robertson et al., 1997), presented on a touchscreen display (7″ diagonal, 800 by 480 pixels resolution) mounted inside the cab of the driving simulator. The SART was included in the experiment to roughly simulate cognitively demanding office work, which could potentially influence participant performance or mind wandering frequency on the second drive of each day via a depletion of executive resources that would otherwise maintain attention towards the primary task (Thomson et al., 2015). However, the purpose of including the SART wasn’t to examine the effect of the SART *per se*, but rather to ensure that enough mind wandering instances occurred throughout the course of the study for comparison of mind wandering and on task states. Within each trial of the SART, participants were presented with a single digit between 1 and 9 on the center of the display. Participants were instructed to click a response button on a Logitech Wireless Presenter remote to the digits 1, 2, 4, 5, 6, 7, 8 or 9, while withholding any response to the remaining digit 3. Participants held the remote with their right hand for the duration of the task. Participants completed two blocks of the task on each day of participation, with each block containing 450 trials and taking approximately 9 min to complete. Each block contained 50 presentations of each of the digits 1 through 9, in a randomized order. Each stimulus remained on-screen for 250 ms, following which it was removed. Response to each SART stimulus were collected for up to 1-s following stimulus onset on each trial. The inter-stimulus interval was jittered with a continuous uniform distribution between 1050 ms and 1250 ms, rounded to the next presentation frame. Stimuli were presented as white digits on a black background, in addition, black and white tracking patterns were displayed in the four corners of the display to allow forward facing camera of the eye-tracker to track the location of the display. Although performed within the cab of the driving simulator for practical purposes, the participants did not perform a drive or interact with the simulator while performing the SART. During performance of the SART the visual displays of the stimulator displayed images of visual noise generated by randomizing the pixels of screenshots of the driving scenario, as used within each day’s pre drive baseline condition. The SART was implemented within the Psychophysics Toolbox (Brainard, 1997; Pelli, 1997; Kleiner et al., 2007) for MATLAB.

### Attentional Probes

Within both the driving scenarios and SART, participants were probed to self-report their current attentional state. Probes were initiated by an auditory tone, composed of a 440 Hz sine wave, 500 ms in length with 10 ms onset and offset ramps, presented at 70 dB SPL via speakers mounted behind the seat of the simulator. Concurrent with tone presentation, a touchscreen mounted on the dashboard of the vehicle presented a response screen displaying two buttons, labeled “Mindwandering” and “On Task”. If participants responded “Mindwandering”, a second screen was presented displaying two buttons labeled “Aware of Mind Wandering before probe” and “NOT aware of Mind Wandering until probe”. The second response was collected as the effects of mind wandering may be particularly pronounced when participants are both off-task and unaware of their inattentiveness (see Smallwood and Schooler, 2015). The time interval to the next attentional probe was relative to the submission of response to the current probe, with a response to stimulus interval jittered with a continuous uniform distribution between 30 s and 90 s. Attentional probes were presented in both the driving task and SART. In the SART, the attentional probes were always presented between SART trials. Prior to participation, participants were explained the probe procedure and response screen, and allowed to familiarize themselves with the attentional probe tone. Attentional probe presentation and response collected was implemented within the Psychophysics Toolbox (Brainard, 1997; Pelli, 1997; Kleiner et al., 2007) for MATLAB.

### Electrophysiology

EEG was recorded using a BrainVision ActiChamp 32-channel active EEG system in conjunction with BrainVision PyCorder (v1.0.8) recording software. All electrode impedances were prepared to below 25 kΩ prior to data collection, the threshold recommended by the EEG system manufacturer for active electrodes. EEG was recorded at a sampling rate of 500 Hz, an online reference of electrode TP9 (left mastoid process), and an online band-pass filter between 0.01 Hz and 100 Hz. Offline, EEG data was processed using MATLAB in conjunction with the EEGLAB toolbox (Delorme and Makeig, 2004). Data was re-referenced to the average of TP9 and TP10 (left and right mastoid process electrodes), low-pass filtered using a filter with 40 Hz cutoff and 10 Hz transition bandwidth, and high-pass filtered using a windowed sinc FIR filter with Blackmann window, 0.1 Hz cutoff, and 0.2 Hz transition bandwidth, both as implemented in the EEGLAB function pop_firws. An additional copy of the data was high-pass filtered at 1 Hz with a 2 Hz transition bandwidth, for independent components analysis (ICA) decomposition (Debener et al., 2010; Winkler et al., 2015). Following filtering, artifactual electrodes were identified and removed via the EEGLAB pop_rejchan function, where artifactual electrodes are defined as those exceeding ±3 standard deviations probability. Artifactual electrodes were identified using the 0.1 Hz high-pass filtered data (which is used for the final analysis), however the same electrodes were subsequently also removed from the 1 Hz high-pass filtered data (which is used for ICA only). Across the 45 sessions within the experiment, an average of 1.29 electrodes were removed (*SD* = 0.82, min = 0, max = 3).

The copy of the data high-pass filtered at 1 Hz was epoched into 1 s intervals, with any 1-s epoch with activity ±500 μV on any electrode removed from further analysis. The remaining epochs were decomposed via ICA, using the Extended InfoMax algorithm as implemented in EEGLAB, following which the ICA weights were copied from the 1 Hz high-pass filtered to the 0.1 Hz high-pass filtered data. Independent components (IC) reflecting eye movements and eye blinks were identified via manual inspection of IC topography and spectra, and subtracted from the data. This ICA procedure was performed for each day of recorded data separately, as the electrodes are unlikely to be located in precisely the same location for the same participant across days.

Following rejection of artifactual ICs, EEG data was epoched into 10 1-s non-overlapping epochs preceding each auditory probe, with each epoch labeled according to the corresponding probe response. Following epoching, any epoch with activity ±100 μV on any electrode was removed from further analysis. Any electrodes previously removed due to artifact were interpolated via spherical interpolation for the purpose of topographic plotting.

For measurement of EEG spectra, the data from each of the remaining 1-s epochs were linearly detrended and converted from the time domain to the frequency domain via Welch’s periodogram method, as implemented in the MATLAB function pwelch. Power values for each frequency bin within each epoch were converted into decibels (dB) power (10 * log10(power)) to more closely approximate a normal distribution. The spectral power preceding each auditory probe was then computed by averaging the dB scaled power from the remaining 1-s epochs preceding that probe. Of interest was activity in the theta and alpha bands of the EEG. *A priori*, electrode Fz was selected for investigation of theta power, while electrode Pz was selected for investigation of alpha power. Electrode Fz was selected for theta activity as it is the electrode where frontal midline theta is most prominent. Electrode Pz was selected for alpha activity as enhanced alpha activity at electrode Pz has been reported when attention is directed internally, such as to the content work working memory, and has previously been reported to be sensitive to lapses of attention to external stimuli (O’Connell et al., 2009). The frequency bins representing the peak theta and alpha frequency across subjects were identified via visual inspection of the spectrum collapsed across all participants and conditions under study at the stated electrodes. The peak theta frequency was identified as the bin centered on 5.85 Hz, while the peak alpha frequency was identified as the bin centered on 8.78 Hz. The dB power within these frequency bins were used for statistical comparison across experimental conditions.

For measurement of time domain EEG activity (ERP), EEG was epoched from −200 ms to 800 ms relative to the onset of the attentional probe, baseline corrected to the mean of the activity within the −200 ms to 0 ms baseline period. Following baseline correction rejection, any epoch with activity ±100 μV on any electrode was removed from further analysis. Any electrodes previously removed due to artifact were interpolated via spherical interpolation for the purpose of topographic plotting. Of interest was activity in the auditory N1 and P3a. *A priori*, electrode Cz was selected for analysis of the auditory N1, a plot of N1 topography collapsed across conditions confirmed a Cz maximal negativity during the time period of the auditory N1. The P3a component classically displays a fronto-central maximum. A plot of the P3a time window collapsed across conditions under study suggested a maximal positivity between electrodes Fz and Cz (near the location of electrode FCz, although this electrode not present in the 32-channel montage used within the present study). For this reason, both electrodes Fz and Cz were selected for analysis of the P3a. Time windows of the auditory N1, and P3a components were identified inspection of the ERP collapsed across all participants and conditions under study at the stated electrodes. The time window of the auditory N1 was identified as 110–160 ms following auditory probe onset. The time window representing the P3a component of the ERP was identified as 200–400 ms following auditory probe onset.

### Synchronization

Data from the equipment used within the study were synchronized via the lab streaming layer (LSL) software library^1^. LSL synchronizes the timestamps between multiple devices and computers via a network connection. Specifically, the LSL library was integrated into the driving simulator in order to synchronize the simulator with the computer responsible for the auditory probes, which utilized the LSL MATLAB library. Parallel port trigger events were additionally sent from the computer responsible for the auditory probes directly to the EEG system.

## Results

**Figure 2:**
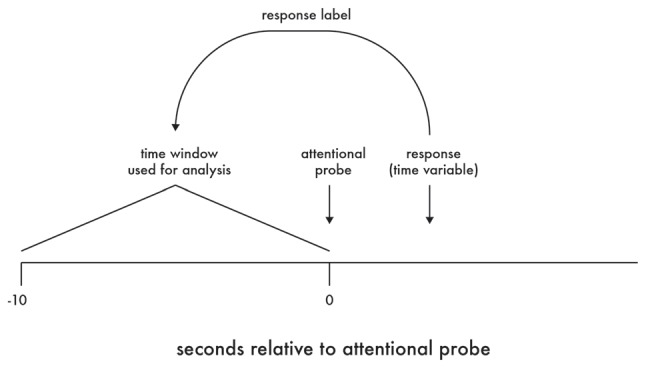
Labeling of data for analysis.

Data reduction was performed using MATLAB, with statistical analyses performed using R (R Core Team, 2015). Specifically, for measures of driver behavior and electrophysiology obtained during each drive, participant attentional state was labeled according to participant response following each attentional probe presentation. Further, the response to each probe was used to label the period of data that had occurred within the 10 s prior to the onset of that probe (see Figure 2).

For data from the driving simulator, measures of mean activity (e.g., speed) and variance (e.g., speed variability) were derived within each 10-s period. For data from the EEG, due to the potential of EEG artifact, each 10-s period was first split into 10, 1-s epochs, with any 1-s period containing artifact removed from further analysis and only the remaining 1-s periods averaged as representative of that 10-s period. As previously noted, attentional probes that occurred within or up to 10-s following a roadway curve were excluded from analysis. Finally, all data were winsorized to ±3 standard deviations from the mean of the corresponding outcome measure (Dixon and Tukey, 1968).

Driver behavior and physiology were analyzed using linear mixed effects modeling. The linear mixed effects analyses were performed using the Linear Mixed-Effects Models using “Eigen” and S4 (lme4) package (Bates et al., 2015) and the Tests in Linear Mixed Effects Models (lmerTest) package (Kuznetsova et al., 2016) for R. As SRR is a count variable (i.e., number of reversals per second), a poisson generalized linear mixed model was analyzed using lme4 and lmerTest. Because participants varied in the number of mind wandering instances reported, Satterthwaite type III approximations were used to calculate the denominator degrees of freedom. The fixed effects for all models included state (mind wandering, on task) and drive (drive one, drive two) as categorical variables, entered as sum contrasts (−1, 1) and day as a continuous variable, mean centered across the data set. Model intercepts were set as a random effect, allowing participants to have varying intercepts.

### Questionnaire Analyses

The descriptive statistics for the questionnaire data obtained on the first day of participation are displayed in Table 1. However, due to the limited sample size, further analyses were not performed.

**Table 1:** Descriptive Statistics (*M*, *SD*) for Questionnaires.

Questionnaire	*M*	*SD*	Range
ARDES	1.70	0.30	1.33–2.22
ARCES	31.44	3.13	29–38
CFQ	39.33	13.98	27–68
MAAS	3.17	0.77	2.07–4.47
MWS	2.91	0.44	2.23–3.54

*Note: ARDES, Attention-Related Driving Errors Scale; ARCES, Attention-Related Cognitive Errors Scale; CFQ, Cognitive Failures Questionnaire; MAAS, Mindful Attention Awareness Scale; MWS, Mind Wandering Scale*.

### SART Performance

The results of the SART are not reported here in depth, however participants attempted to perform the task, correctly responding to over 99% of the non “3” stimuli on average, and correctly withholding response to around 60% of the “3” stimuli, on average.

### Prevalence of Mind Wandering

Responses to the attentional probes were analyzed to identify any changes in the frequency of mind wandering, or the frequency of aware relative to unaware mind wandering, over day or drive. The frequency of mind wandering, as well as the frequency of mind wandering awareness, were tested across days and drives via generalized linear mixed effects models with logit link function, using the lme4 (Bates et al., 2015) and lmerTest (Kuznetsova et al., 2016) packages for R. For mind wandering frequency, the effect of drive reached significance, such that participants were more likely to respond “mind wandering” during the second drive (*z* = 2.36, *β* = 0.16, *p* = 0.019) relative to the first drive. Neither the effect of day, nor the day by drive interaction reached significance for mind wandering frequency. For mind wandering awareness frequency, the effect of day reached significance, such that participants were more likely to be aware of their mind wandering as days of the experiment progressed, *z* = 4.22, *β* = 0.23, *p* < 0.001. Neither the effect of drive, nor the drive by day interaction reached significance for mind wandering awareness frequency.

**Figure 3:**
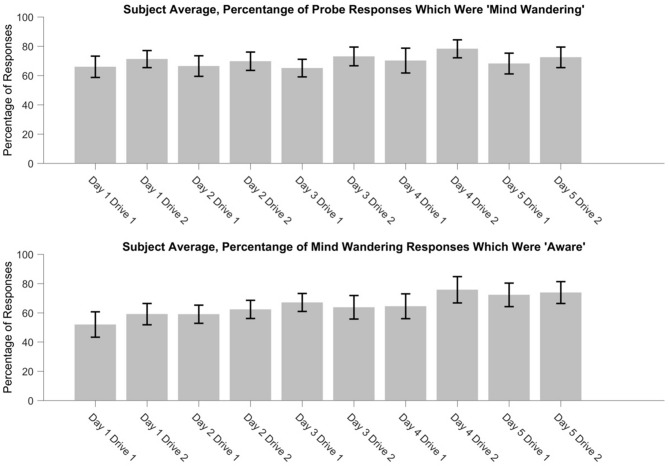
The percentage of attentional probes in which participants responded “mind wandering” (top), and the percentage of “mind wandering” responses that were subsequently categorized as “aware” (bottom), over days and drives.

For illustrative purposes, the percentage of mind wandering episodes were identified by computing the percentage of probes that were responded to as “mind wandering” during each drive (“mind wandering”)/(“mind wandering” + “on task”). Mind wandering awareness percentage was identified by computing the percentage of “mind wandering” responses that were subsequently responded to as “aware”, during each drive (“aware mind wandering”)/(“aware mind wandering” + “unaware mind wandering”). In general, participants often self-reported that they were mind wandering (see Figure 3). Collapsing across day of participation and drive within day, participants responded “mind wandering” for 70.10% of probe responses (*SD* across participants = 17.00%). Additionally, participants often self-reported they were aware that they were mind wandering at the time of the probe. Collapsing across day of participation and drive within day, participants responded that they were aware of their mind wandering for 65.00% of mind wandering responses (*SD* across participants = 16.54%).

### Driving Behavior

Data from the driving simulator, including speed, lane offset and steering wheel rotation were recorded at 30 Hz. Within each 10-s window, the raw data from the simulator was used to derive speed variability, lateral position variability, lane deviation and the SRR. Lateral position variability was defined as the vehicle’s lateral position standard deviation (SDLP) in meters. Lane deviation was defined as the root mean square of the distance in meters from the center of the lane. Lastly, SRR was measured as the number of reversals per second (Hz) when steering angle passed through zero with a degree offset greater than or equal to 2 (He et al., 2014). Since SRR is a count variable requiring a Poisson analysis, it was necessary to multiply all values by 10 for this analysis. Additionally, logarithmic transformations [log10 (x) + 3] were performed for speed variability, lateral position and lane deviation to account for positively skewed data. The constant of 3 was added to the logarithmically transformed variables to shift the output scale back to a positive range, for convenience.

**Figure 4:**
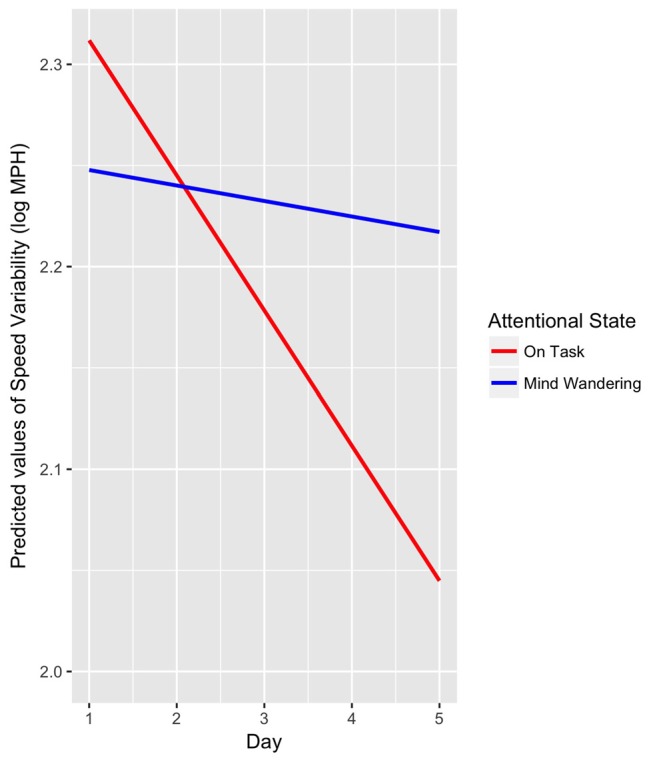
Linear mixed model interaction between attentional state (“mind wandering”, “on task”) and days (1–5) as predictors of speed variability, *p* = 0.009.

The results of the linear mixed model for speed variability showed that drive (1, 2) was not a significant predictor (drive 1: *M* = 0.27, *SE* = 0.017; drive 2: *M* = 0.26, *SE* = 0.018), *p* = 0.84. There was a significant interaction between attentional state and day such that, speed variability decreased during “on task” periods across days, *t*_(1158)_ = −2.61, *β* = −0.03, *p* = 0.009 (see Figure 4).

**Figure 5:**
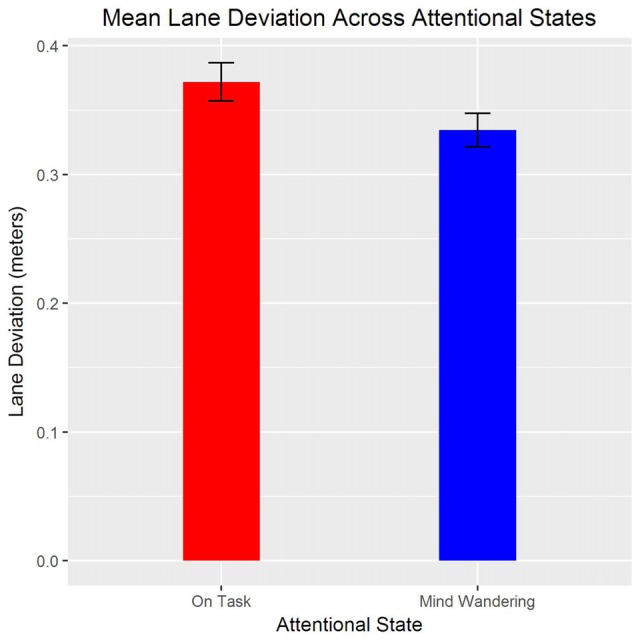
Means and standard errors of lane deviation for “mind wandering” and “on task” attentional states.

Additionally, lane deviation was significantly greater for drive 2 (*M* = 0.36, *SE* = 0.015) compared to drive 1 (*M* = 0.34, *SE* = 0.013), *t*_(1157)_ = −2.05, *β* = −0.02, *p* = 0.041. There was also a significant effect for state such that, lane deviation was greater during “on task” compared to “mind wandering” attentional state, *t*_(1164)_ = 2.58, *β* = 0.02, *p* = 0.01 (see Figure 5). However, day did not significantly predict lane deviation, and there were no significant interactions, *p*s > 0.05.

**Figure 6:**
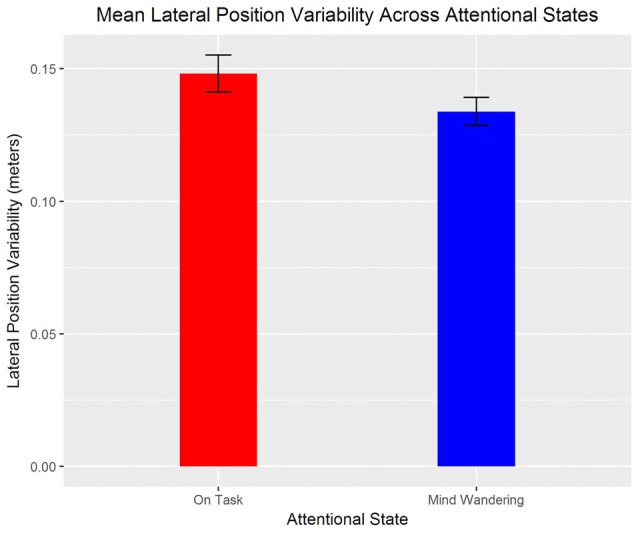
Means and standard errors of the standard deviation of lateral position (SDLP) for “mind wandering” and “on task” attentional states.

SDLP was also significantly greater when “on task” compared to “mind wandering”, *t*_(1165)_ = 2.07, *β* = −0.02, *p* = 0.04 (see Figure 6). There was also a significant interaction between drive and day such that, SDLP significantly increased for the second drive across days, *t*_(1157)_ = 2.36, *β* = 0.02, *p* = 0.02.

**Figure 7:**
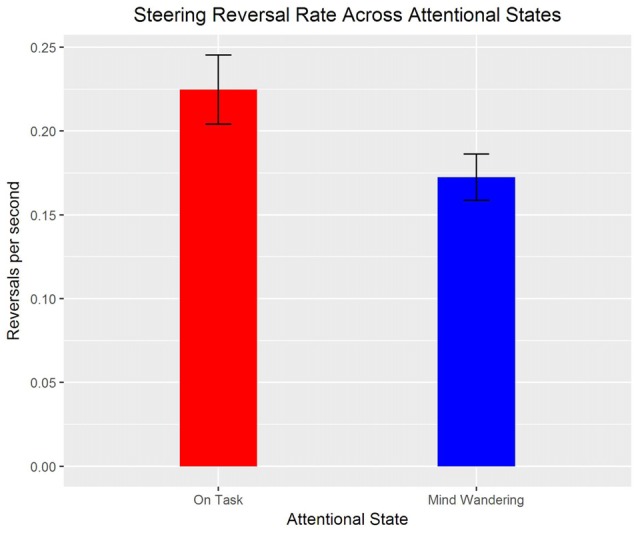
Means and standard errors of steering reversal rate (SRR; per second) for “mind wandering” and “on task” attentional states.

A poisson mixed model showed that SRR was also significantly greater when “on task” compared to “mind wandering”, *z* = 6.77, *β* = 0.16, *p* < 0.001 (see Figure 7). However, day and drive did not significantly predict SRR, and there were no significant interactions, *p*s > 0.05.

### EEG Spectra

**Figure 8:**
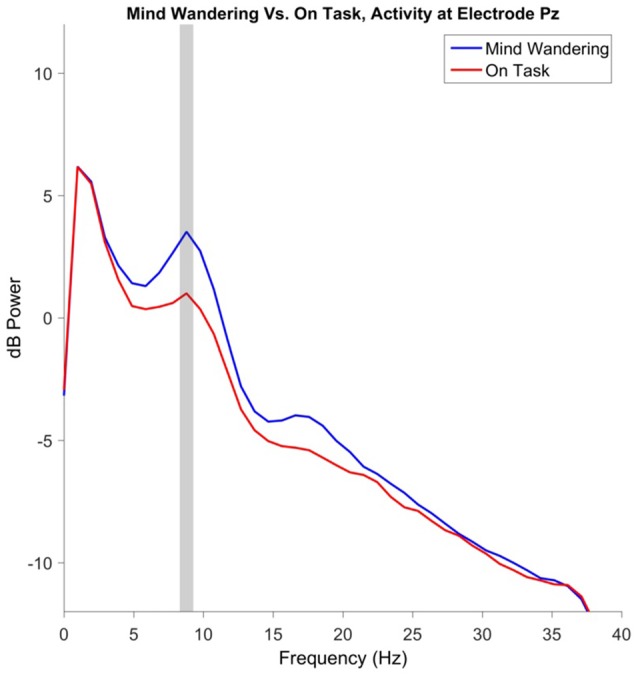
Electroencephalography (EEG) power spectrum at parietal electrode site Pz, preceding “mind wandering” vs. “on task” responses. The shaded gray region represents the width of the frequency bin defined as alpha, identified via inspection of the grand mean of both “mind wandering” and “on task” conditions.

**Figure 9:**
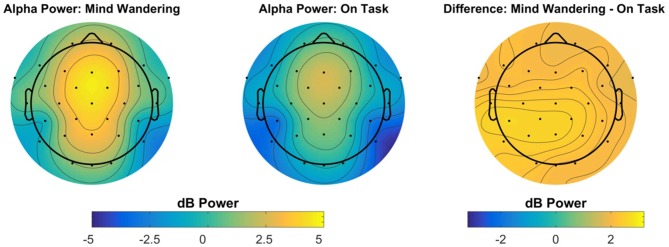
Topography of alpha power preceding “mind wandering” vs. “on task” responses. The right-most panel represents the topography of the difference between “mind wandering” and “on task” responses.

Power in the theta frequency at frontal electrode Fz and power in the alpha frequency at parietal electrode Pz were selected *a priori* for analysis. No effects reached significance for frontal theta power. Alpha power was increased during “mind wandering” periods relative to “on task” periods, *t*_(1146.4)_ = 4.41, *β* = 0.43, *p* < 0.001. Additionally, alpha power increased over days of participation, *t*_(1144)_ = 3.36, *β* = 0.22, *p* < 0.001. The main effect of alpha power at Pz is illustrated in Figure 8, with the topography illustrated in Figure 9.

### ERP to Probe Tone

**Figure 10:**
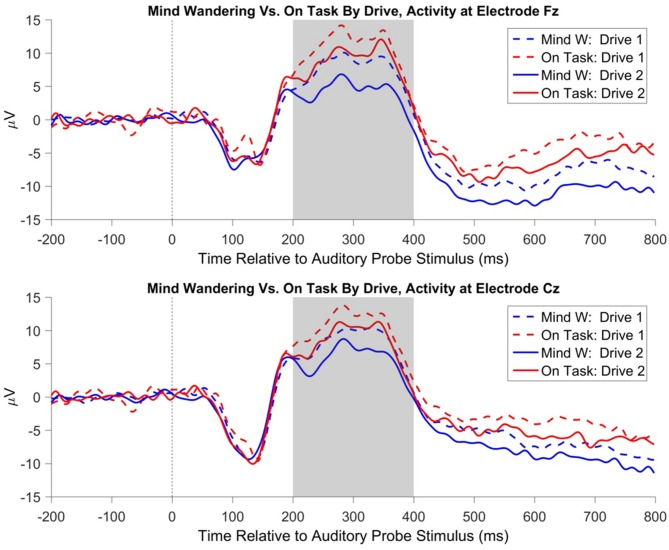
Event related potential (ERP) at electrode Fz to auditory probe tones, separated by participant response and the drive number in which the probe tone occurred. The shaded gray region represents the time-window used to evaluate P3a amplitude. Probe tones presented during periods in which participants responded “on task” have a greater magnitude of P3a relative to probes presented during periods in which participants responded “mind wandering”. In addition, probe tones presented during the first drive of the day have a greater magnitude of P3a relative to probes presented during the second drive of the day.

**Figure 11:**
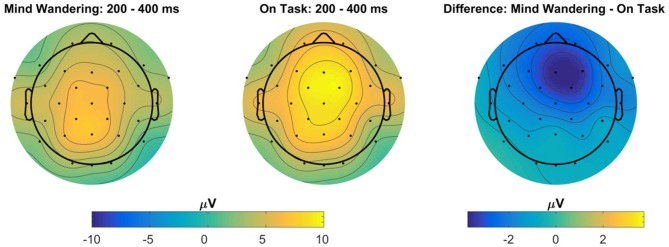
Topography of the ERP to the auditory probe tone, for “mind wandering” vs. “on task” responses, averaged within the 200–400 ms P3a window following probe onset, collapsed across all days and drives. The right-most panel represents the topography of the difference between “mind wandering” and “on task” responses.

ERPs to the onset of the auditory probe were analyzed in order to determine whether the auditory probes were processed differently with respect to subjective attentional state. The auditory N1 component was analyzed at electrode Cz, however no main effects or interactions reached significance. The P3a component was analyzed independently at electrodes Fz and Cz. For both electrode locations, the P3a component in response to the auditory probe was reduced in magnitude for probes which were subsequently responded to as “mind wandering” relative to probes which were subsequently responded to as “on task”, electrode Fz: *t*_(1115)_ = −3.64, *β* = −1.45, *p* < 0.001, electrode Cz: *t*_(1115)_ = −2.84, *β* = −1.09, *p* = 0.005. In addition, for both electrode locations, there was a significant effect of drive, such that the P3a during the second drive was diminished relative to the first, electrode Fz: *t*_(1108)_ = −3.27, *β* = −1.24, *p* = 0.001, electrode Cz: *t*_(1108)_ = −2.48, *β* = −0.91, *p* = 0.013. No effects of day of participation, nor any interactions, reached significance for the P3a at either electrode location. The main effects of attentional state and drive on P3a magnitude are illustrated in Figure 10. The topography of the effect of attentional state is illustrated in Figure 11.

## Discussion

The experimental design was intended to roughly simulate drives to and from work, separated by a cognitively depleting work task. In this study, participants drove the same highway route twice a day for 5 days. Between the two drives on a given day, participants completed a task requiring sustained attention, the SART. Participants self-reported their current attentional state, indicating whether they were either “on-task” or “mind wandering”, in response to periodic probe tones. Additionally, following mind wandering responses, participants indicated whether they were aware or not aware of their mind wandering prior to the attentional probes.

The driving scenarios were designed to be rather repetitive and monotonous in order to increase the incidence of mind wandering (Berthié et al., 2015; Thomson et al., 2015) and ensure that enough instances of mind wandering would be generated for study. On average, 70.10% of the probes in the present study were responded to as “mind wandering” by the study participants. In contrast, Killingsworth and Gilbert (2010) report the results of a study probing individuals as to the content of their thoughts throughout everyday life outside the laboratory, reporting that participants respond that they are thinking about something other than what they are currently doing approximately 47% of the time. The high frequency of mind wandering in the present experiment would likely be lessened if the driving scenarios were made to be more demanding. For example, Lin et al. (2016) report a study measuring EEG activity in a motion-base driving simulator, asking participants to detect lane departures using either visual and motion information (the simulator could move in response to road conditions such as rumble strips), or only visual information (the simulator’s motion capabilities were deactivated). The authors report less DMN activity when participants were performing the more demanding version of the task without motion information. The addition of ambient traffic, or a more complex navigation task that also required navigation would also likely decrease the prevalence of self-reported mind wandering.

With consideration of previous work that has suggested increases in driver inattention as route familiarity increases (Yanko and Spalek, 2014), it was expected that mind wandering frequency might increase over the 5 days of participation. Instead, mind wandering frequency did not significantly differ over days of participation, though mind wandering did increase for the second drive relative to the first drive within the same day of participation. It is possible that the repetitive nature of the drives induced a maximum amount (or ceiling effect) of mind wandering while still being able to safely operate the simulated vehicle, thereby reducing the potential to see increased mind wandering frequency across days. The SART was performed between drives to roughly simulate performing a work task between commutes, and to ensure that enough mind wandering instances were available for study. As all participants performed the SART, we cannot comment on whether the increase in mind wandering frequency in the second drive of the present study is due to increased route familiarity, resource depletion from the SART, or a time on task effect.

Participants became significantly more aware that they were mind wandering across days. Specifically, for probe responses that were labeled mind wandering, there was a significant increase over days of participation in the frequency that participants reported that they were “aware” of their mind wandering at the time of the probe. It should be noted that the work of Yanko and Spalek (2014) did not query participant subjective state, and instead measured participant inattention by assessing their ability to detect hazards in the environment. The present experiment, in contrast, did ask participants to report their subjective attentional state, but did not include roadway hazards. Thus the present results may not contradict Yanko and Spalek (2013) but instead suggest a more complex relationship between driving performance and participant awareness of their attentional state. In the present experiment, when examining the 10-s periods preceding the attentional state probes, EEG alpha power increased over days of participation, in addition to more generally being elevated during periods of mind wandering. Taken together, it is possible that participants did become somewhat less attentive to the driving environment over days of participation in the present study, despite the lack of increase in subjective mind wandering reports over days of participation.

Power in the alpha band of the EEG was of greater magnitude during periods of self-reported mind wandering, relative to periods of self-reported on-task performance. Greater alpha power during periods of task inattention has also been reported for a variety of non-driving tasks. For example, O’Connell et al. (2009) report that within the continuous temporal expectancy task (CTET), the magnitude of alpha power over parietal electrodes is elevated prior to trials in which participants missed the target of interest, relative to trials in which participants correctly detected the target. Importantly, the increase in alpha band activity for miss trials was detectable up to 20 s prior to trial onset, suggesting that a slow modulation of top-down control contributes to lapses of attention within the CTET. Alpha power has further been related to inattention within a driving context more specifically. Within a driving simulator task in which participants were provided auditory notifications of lane departures, notifications that were behaviorally successful were associated with decreased alpha power following the notification, while for ineffective notifications alpha power remained elevated (Lin et al., 2013). Although consistent with other work on lapses of attention, an increase in alpha power during mind wandering is in contrast to what has been reported for self-detected mind wandering in a breath-counting task, wherein alpha power is suppressed immediately prior to self-detected mind wandering (Braboszcz and Delorme, 2011). This apparent difference may be resolved by considering that the alpha oscillation may serve a different role within the primary task used within the present study, simulated driving, in comparison to the primary task used within Braboszcz and Delorme (2011), eyes closed breath-counting. Future exploration of the possibility of using alpha power as a potential predictor of the mind wandering state, and thus a greater probability of missing a potentially hazardous event warrant further research. A supplementary method of examining a possible predictive potential for participants to miss critical events can be found in analysis of the ERPs.

Participants were periodically presented with an auditory tone, notifying them to indicate their current attentional state. Analysis of the ERP to this probe tone suggested that the P3a component was larger in response to tones that were subsequently responded to as “on task”, relative to tones that were subsequently responded to as “mind wandering”. As the P3a component is thought to reflect the orienting of attention towards a novel stimulus (Polich, 2007, 2011), this result supports the decoupling hypothesis of mind wandering (Smallwood, 2011) and is suggestive that participant attention towards the external environment was diminished during periods that were subjectively labeled as “mind wandering” relative to “on task”. Despite previous reports that mind wandering modulates the amplitude of early sensory components of the ERP (Baird et al., 2014; Broadway et al., 2015), we did not observe any statistically significant modulation of the auditory N1 component to the probe tones by attentional state. As early sensory components are known to be modulated by attention (Luck et al., 2000), the lack of an effect on the auditory N1 may reflect a lack of top-down attention towards the auditory stimulus in either the mind wandering or on task state. The auditory tone in the present experiment was supra-threshold, unpredictable with respect to onset, and did not require discrimination, only simple detection in order to provoke an attentional probe response. This is in contrast to influences of mind wandering on early sensory components in previous reports, for which the ERP is time-locked to a primary task stimulus (Baird et al., 2014; Broadway et al., 2015).

There are several limitations that should be noted. Our participants were restricted to young (18–29 years) individuals free from disease and other visual or health impairments that might compromise driving. It is not known whether mind wandering might manifest similarly in an older population or in individuals with certain health disorders. Further, none of our participants were shift workers and therefore it is unknown whether disruptions in circadian rhythms might impact performance or the underlying physiology. Additionally, the results were obtained in a driving simulator under carefully controlled conditions. More variability in both environment and behavior could be expected under naturalistic driving conditions.

In summary, both driving behavior and EEG activity demonstrated sensitivity off-line to distinguishing between periods of self-reported mind wandering vs. being on task. These results are largely in line with previous studies on mind wandering during driving, and on attentional processes as assessed with EEG, and support that mind wandering has an impact on both driving performance and the driver’s underlying physiology. Future work could extend these results by examining more closely a driver’s reaction to potential hazard situations when mind wandering vs. alert. Drivers may be expected to be less likely to react appropriately to a potential hazard (particularly if it occurs in peripheral vision since during mind wandering since gaze is narrowly focused more centrally). Further, the current results suggest drivers may be less likely to detect an auditory or visual warning while mind wandering. Future work should examine the potential for advanced auditory warnings to aid hazard mitigation in differing attentional states.

## Author Contributions

CLB, DMR, DB, JDL, NL and JSH contributed to the design of the experiment, DMR and DB collected and analyzed the data. CLB, DMR, DB, NL and JSH contributed to the writing of the manuscript.

## Conflict of Interest Statement

The authors declare that the research was conducted in the absence of any commercial or financial relationships that could be construed as a potential conflict of interest.

## Footnotes

^1^https://github.com/sccn/labstreaminglayer
